# The Single Nucleotide Polymorphism rs1014290 of the* SLC2A9* Gene Is Associated with Uric Acid Metabolism in Parkinson's Disease

**DOI:** 10.1155/2017/7184927

**Published:** 2017-10-11

**Authors:** Jiangfang Miao, Jing Liu, Li Xiao, Jiedi Zheng, Chunfeng Liu, Zufu Zhu, Kai Li, Weifeng Luo

**Affiliations:** ^1^Jiangsu Key Laboratory of Translational Research and Therapy for Neuro-Psycho-Diseases and The Second Affiliated Hospital of Soochow University, Soochow University, Suzhou, Jiangsu 215021, China; ^2^Jiangsu Jiangyin People's Hospital, Jiangyin, Jiangsu 214400, China; ^3^College of Pharmaceutical Science, Soochow University, Suzhou, Jiangsu 215123, China

## Abstract

Individuals with Parkinson's disease (PD) have lower uric acid levels than those without PD, and the CC genotype and C minor allele of a single nucleotide polymorphism (SNP), rs1014290 of* SLC2A9*, are associated with lower uric acid levels. We investigated the association of rs1014290 with uric acid metabolism in a cohort of PD cases (220) and controls (110) in a Han Chinese population. Uric acid levels were determined and rs1014290 was assayed using a mutation-sensitive on/off switch technology. PD uric acid levels (291.65 ± 76.29 *μ*mol/L) were significantly lower than the controls (325.73 ± 74.23 *μ*mol/L, *P* < 0.001, *t*-test). Individuals with rs1014290 TT and CT genotypes had higher uric acid levels, and those with the CC genotype had the lowest uric acid levels among both control and PD cases. The CC genotype and the C minor allele were statistically more frequent in the PD group compared to the control group. Those with the CC genotype had a statistically significant higher risk of PD than those with the TT or TC genotype (odds ratio [OR] = 2.249, 95% confidence interval [CI]: 1.129–4.480, and *P* = 0.021). Thus, SLC2A9 rs1014290 is related to lower uric acid levels in PD patients and can be a risk factor for PD in the Han population.

## 1. Introduction

Parkinson's disease (PD) is one of the most common neurodegenerative diseases, characterized by the extrapyramidal symptoms of resting tremor, rigidity, bradykinesia, and postural instability. Males have a significantly higher incidence rate of PD, approximately 1.5 times greater than females [1]. Although the etiology of PD remains unclear, it is generally considered to be a combination of complex genetic and environmental factors [2]. Several epidemiological studies suggest that high plasma uric acid levels decrease the risk of developing PD [3, 4], and serum uric acid can be a predictor of clinical and radiographic progression in PD [5].

*SLC2A9* is considered to be the most effective urate transporter [6], affecting circulating uric acid levels. The single nucleotide polymorphism (SNP) rs1014290 of the* SLC2A9 *gene has been reported to influence the age of onset of PD [7]. Here, through a retrospective case-controlled study, we aimed to identify the correlation between* SLC2A9* SNP rs1014290 and PD pathogenesis and to explore whether this relationship was affected by uric acid metabolism in the Han Chinese population. In all, we aimed to find genotype-endophenotype-exophenotype relationships among rs1014290 in* SLC2A9*, serum uric acid level, and PD in a select Han Chinese subpopulation.

## 2. Methods

### 2.1. Participants

A total of 220 patients with sporadic idiopathic PD from the Neurology Department, Second Affiliated Hospital of Soochow University in China, were selected from January 2011 to November 2012. The PD diagnosis was made by neurologists based on criteria consistent with the UK PD Society (UKPDS) Brain Bank criteria [8]. Sporadic idiopathic PD is defined as one without a family history of PD. For comparison, 110 unrelated Han healthy controls matched in gender, age, and area of residence were included and investigated by our research group during the PD epidemiological study. All healthy controls were examined by neurologists to rule out neurodegenerative disorders, and detailed clinical data was recorded. And those who had gout and other diseases, such as liver and kidney diseases, were ruled out in all subjects. All subjects provided written informed consent. Ethics approval was obtained from the ethics committee of the Second Affiliated Hospital of Soochow University in China. 4 ml of venous blood was extracted from all subjects in the fasting state, 2 ml for testing serum uric acid levels and 2 ml for gene detection.

### 2.2. Uric Acid Levels Testing

Uric acid levels were determined using a Beckman Coulter (USA) automatic biochemical analyzer with the original kits at the Second Affiliated Hospital of Soochow University.

### 2.3. Genotyping

**Figure 1:**
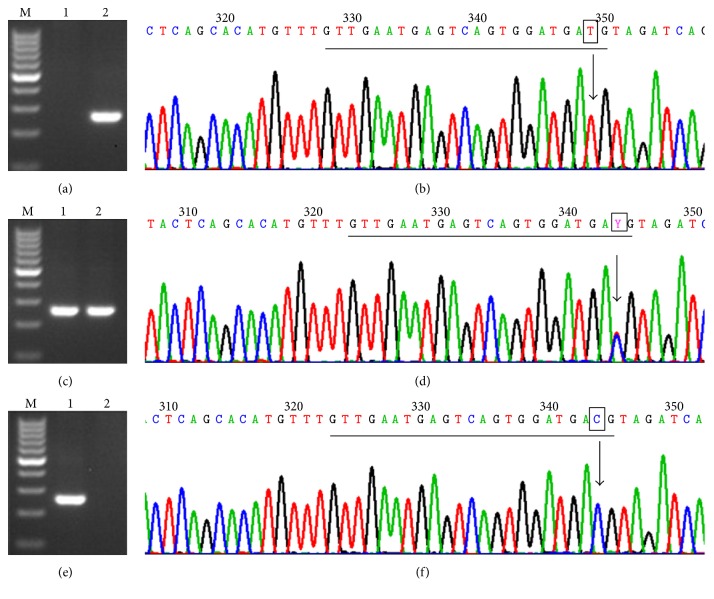
Representative illustrations of genotyping data and sequencing chromatography results. PCR product from the C allele template (lane 1) and the amplified fragment from the T allele-specific DNA using the on/off switch (lane 2). (a, c, and e) are the relevant genotypes confirmed by Sanger sequencing. The corresponding genotypes are CC, CT (Y), and TT as indicated with the box. M, marker.

DNA was extracted from peripheral blood using the Magman TM Blood DNA Kit (GenoTheramics, USA) based on magnetic beads. Genotyping for rs1014290 was performed using a new assay employing proofreading PCR technology. We assayed SNP genotyping using a mutation-sensitive on/off switch consisting of 3′ terminal phosphorothioate-modified allele-specific primers and high-fidelity exo+ DNA polymerases. In recent years, this novel technology has been successfully employed in genetic analysis [9–11]. The mutation-sensitive on/off switch eliminates false positive errors and significantly decreases false negatives, which has been demonstrated to have a higher specificity than assays mediated by low-fidelity DNA polymerase. During the assay development stage, Sanger sequencing was used to confirm the proofreading PCR results (Figure 1).

### 2.4. Statistical Analyses

All statistical analyses were performed using IBM SPSS Statistics 23. The Hardy-Weinberg equilibrium (HWE) of* SLC2A9* rs1014290 for PD patients and controls was verified by Pearson's Chi-Square test, and “deviation” from Hardy-Weinberg is being tested. Allele frequency and genotype frequency between the two groups were compared with Chi-Square test. Uric acid levels were used to compare between the two groups using independent sample *t*-test. The odds ratio (OR) and 95% confidence interval (CI) were estimated using Chi-Square tests to examine the CC genotype frequency and minor allele frequencies for rs1014290 between PD patients and controls.

## 3. Results

### 3.1. Clinical Characteristics of Study Participants

This study included 220 patients with idiopathic PD and 110 unrelated controls, of which 54.5% were male. There was no difference between the mean ages of PD patients (68.10 ± 9.55 years) and those of controls (69.48 ± 8.23 years, *P* = 0.175, *t*-test). The mean serum uric acid concentration of PD patients (291.65 ± 76.29 *μ*mol/L) tended to be lower than that of the controls (325.73 ± 74.23 *μ*mol/L, *P* < 0.001, *t*-test) (Table 1).

### 3.2. The rs1014290 C Allele Genotype Was More Prevalent in the PD Group Compared to the Control Group

The genotype frequencies of the PD group and the control group were in agreement with the HWE (PD: *χ*^2^ = 1.318, *P* > 0.05; controls: *χ*^2^ = 0.689, *P* > 0.05, Pearson Chi-Square test). The minor allele frequency of rs1014290 was 52.0% for the PD group and 42.7% for the control group, which were statistically significant (*χ*^2^ = 5.347, *P* = 0.021, OR = 0.681, and 95% CI = 0.492–0.944, Pearson Chi-Square test) (Table 2). The CC genotype frequencies of rs1014290 were 30.0% for the PD group and 15.5% for the control group (*χ*^2^ = 8.242, *P* = 0.004, OR = 0.427, and 95% CI = 0.236–0.771, Chi-Square test) (Table 2). We then performed multiple factors logistic regression analysis of rs1014290 genotypes on PD risk and found that individuals with the CC genotype had a higher risk of PD than individuals with the TT or TC genotype (OR = 2.189, 95% CI = 1.095–4.376, and *P* = 0.027) (Table 3).

### 3.3. Clinical Characteristics of PD Patients

The mean onset age of PD patients with the rs1014290 CC genotype (59.24 ± 9.77 years) was lower than that of those with the TT + TC genotype (63.21 ± 9.51 years, *P* = 0.005, *t*-test). For detailed data of clinical characteristics of PD patients please see Table 1.

### 3.4. Genotype-Dependent Serum Uric Acid Levels

The mean serum uric acid concentration of individuals with the CC genotype was lower than that of those with the TT and CT genotypes in both the control (*P* < 0.001, *t*-test) and PD (*P* < 0.001, *t*-test) groups (Table 4).

## 4. Discussion

Uric acid is a strong antioxidant in the human body, having a protective effect against oxidative stress and free radical damage, and may slow the progression of many degenerative nervous diseases [12–14]. Uric acid levels in the brain spinal fluid have a linear relationship with levels found in the blood. The protective effect of high levels of uric acid was first reported by Simon and Vanvunakis in 1964 [15]. Thereafter, several epidemiological studies found that high serum uric acid levels were associated with later onset of PD in males or alleviation of the severity of PD in recent years [4, 16, 17]. Genetic engineering technology has been used to dissect the relationship between uric acid levels in the brain and the development of PD [18], and similar data was gleaned from autopsies performed on patients with PD [19]. As all causal genes dissected from PD genetics account for only approximately 5% of PD cases with familial inheritance, confirmation of the large number of modulation genes of PD has several clinical impacts.

SLC2A9 is the most effective one of all uric acid transporters, and* SLC2A9* variants have been shown to influence circulating uric acid levels. SLC2A9's involvement in uric acid metabolism has been documented by both clinical and animal studies [6, 20, 21]. It is proved that the SLC2A9 variant rs3733591 served as an important genetic checkpoint for tophaceous gout and increased uric acid levels from two geographically diverse populations [22]. Various mutations of SLC2A9 were found in hypouricemia patients: Ile118HisfsX27 in a Czech family [23], exon 7 deletion in Ashkenazi Jewish [24], and R380W and R198C in Japanese [25]. Among the large number of genes related to uric acid metabolism, the present study clearly illustrated the close relationship between rs1014290 genotypes in* SLC2A9* and serum uric acid levels; individuals with the TT and CT genotypes have higher uric acid levels, and those with the CC genotype have the lowest uric acid level. This strong relationship between rs1014290 genotypes in* SLC2A9* and uric acid level phenotype was observed in the normal controls of our present study. Furthermore, we found that PD patients with TT and CT genotypes had higher uric acid levels than those with the CC genotype. The genotype-endophenotype relationship of rs1014290 to the serum uric acid level was also observed in the PD group. Our present study demonstrated that the mean serum uric acid concentration of PD patients was lower than that of the controls, which is consistent with our previous study [26]. We found that, compared with the control group, the rs1014290 minor allele and CC genotype frequencies were higher in the PD group. Furthermore, multiple factors logistic regression analysis showed that individuals with the CC genotype had a statistically significant higher risk of PD than individuals with the TT or TC genotype. The present study has established a link between rs1014290 and PD in a relatively large group of Han Chinese. We suggest that this PD phenotype related to the rs1014290 genotype is possibly mediated through the endophenotype of uric acid metabolism.

The mean onset age of PD patients with the CC genotype was relatively low, which confirms that the CC genotype influences the age of onset of PD [7]. Therefore, there is a genotype-phenotype association of* SLC2A9* rs1014290 with PD through uric acid metabolism in Han Chinese. The polymorphism of rs1014290 in* SLC2A9* may be a risk factor for PD in the Han Chinese population.

Uric acid levels of PD patients with TT and CT genotypes were significantly reduced compared to the controls. Low serum uric acid levels in PD patients were associated not only with the polymorphism of rs1014290 in* SLC2A9* loci, but also with other factors. Various SNPs near SLC2A9 have shown to be linked to uric acid level, such as SLC17A1, SLC22A11, and SLC22A12 [27].

Based upon these data, we suggest that SLC2A9 can be used as either a new drug target or a target for gene therapy. In addition to the potential application in the development of new therapies for PD, the confirmed relationship between rs1014290 and PD may also have diagnostic implications. As rs1014290 in* SLC2A9* is only a modulation factor, its value in the diagnosis and prediction of relative risk to a particular individual may be limited. However, when genotyping is combined with measurement of serum uric acid, high risk or susceptible individuals could be identified. In fact, by further including family history, a more informed assessment could be provided. And the study sample of our study was relatively small; we should collect more samples for further analysis, as larger sample sizes would have allowed further subanalyses and conditional analysis of SNPs in moderate-to-high link with rs1014290 near SLC2A9.

## 5. Conclusions and Future Directions

In conclusion, the present study bridged the divide between genotype, endophenotype, and exophenotype through analysis of rs1014290, serum uric acid level, and incidence of PD. We further confirmed that relatively low uric acid level is a risk factor for PD pathogenesis and that the rs1014290 of* SLC2A9* can be a risk factor for PD in the Han population. The protective effect of high serum uric acid level has been reinforced by our study. As the genotyping assay developed by this study is simple, cost-effective, and extremely reliable, its wide application may be beneficial to PD patients for screening purposes as well as individualized medicine.

## Figures and Tables

**Table 1:** Clinical characteristics of study participants. The mean onset age of PD patients with the rs1014290 CC genotype was lower than that of those with the TT + TC genotype.

Group	PD		*P*1	Controls		*P*2
	CC	TT + CT		CC	TT + CT	
Number (M/F)	66 (37/29)	154 (84/70)		17 (8/9)	93 (52/41)	
Age (years)	65.48 ± 9.62	69.22 ± 9.33	0.009	69.59 ± 4.93	69.46 ± 8.72	0.933
Age at onset (years)	59.24 ± 9.77	63.21 ± 9.51	0.005			
Course of disease	6.24 ± 4.00	6.01 ± 4.05	0.699			
UPDRS motor scale	23.21 ± 10.82	23.12 ± 10.88	0.956			
H-Y score	2.00 ± 0.81	2.05 ± 0.86	0.714			

**Table 2:** Frequency of rs1014290 in PD patients and controls in a Han subpopulation. The frequency of the natural CC genotype is significantly higher in PD patients as compared to the controls, and less TT and CT genotypes are observed in PD patients. Analysis of C and T alleles revealed significant differences between groups.

Group	Number	Genotype frequency (%)			*P*	Allele frequency (%)		*P*
		TT	CT	CC		T	C	
PD	220	56 (25.5)	98 (44.5)	66 (30.0)	<0.001	210 (47.7)	230 (52.3)	<0.001
Controls	110	33 (30.0)	60 (54.5)	17 (15.5)		126 (57.3)	94 (42.7)	

**Table 3:** Multiple factors logistic regression analysis of the rs1014290 genotype in *SLC2A9* on the risk of PD^*∗*^. Individuals with the CC genotype in *SLC2A9* had a higher risk of PD than individuals with the TT or TC genotype.

	*B*	SE	Wald	*v*	*P*	OR	95% CI	
							Lower	Upper
TT			6.579	2	0.037			
TC	−0.006	0.274	0.000	1	0.983	0.994	0.581	1.702
CC	0.783	0.354	4.909	1	0.027	2.189	1.095	4.376

^*∗*^Adjusted for age, sex, height, and body weight.

**Table 4:** Average serum uric acid levels in the PD and control groups. Individuals and PD patients carrying the CC genotype had lower uric acid levels as compared to those with the TT and CT genotypes.

Group	Number	UA (*µ*mol/L)		*P*
		TT + CT	CC	
PD	220	303.50 ± 83.62	264.00 ± 45.22	<0.001
Controls	110	334.14 ± 77.50	279.71 ± 18.32	<0.001
*P*		0.004	0.031	

*P* values compare individuals with different genotypes within the same group; UA, uric acid.
